# Low-Latency Short-Packet Transmission over a Large Spatial Scale

**DOI:** 10.3390/e23070916

**Published:** 2021-07-19

**Authors:** Lei Huang, Xiaoyu Zhao, Wei Chen, H. Vincent Poor

**Affiliations:** 1 Beijing National Research Center for Information Science and Technology (BNRist), Department of Electronic Engineering, Tsinghua University, Beijing 100084, China; huang-l17@tsinghua.org.cn (L.H.); xy-zhao16@mails.tsinghua.edu.cn (X.Z.); wchen@tsinghua.edu.cn (W.C.); 2 Department of Electrical and Computer Engineering, Princeton University, Princeton, NJ 08544, USA

**Keywords:** 6G, short-packet transmission, URLLC, finite-blocklength coding, large spatial scale, relaying, propagation delay, end-to-end delay, decoding delay, asymptotic analysis

## Abstract

Short-packet transmission has attracted considerable attention due to its potential to achieve ultralow latency in automated driving, telesurgery, the Industrial Internet of Things (IIoT), and other applications emerging in the coming era of the Six-Generation (6G) wireless networks. In 6G systems, a paradigm-shifting infrastructure is anticipated to provide seamless coverage by integrating low-Earth orbit (LEO) satellite networks, which enable long-distance wireless relaying. However, how to efficiently transmit short packets over a sizeable spatial scale remains open. In this paper, we are interested in low-latency short-packet transmissions between two distant nodes, in which neither propagation delay, nor propagation loss can be ignored. Decode-and-forward (DF) relays can be deployed to regenerate packets reliably during their delivery over a long distance, thereby reducing the signal-to-noise ratio (SNR) loss. However, they also cause decoding delay in each hop, the sum of which may become large and cannot be ignored given the stringent latency constraints. This paper presents an optimal relay deployment to minimize the error probability while meeting both the latency and transmission power constraints. Based on an asymptotic analysis, a theoretical performance bound for distant short-packet transmission is also characterized by the optimal distance–latency–reliability tradeoff, which is expected to provide insights into designing integrated LEO satellite communications in 6G.

## 1. Introduction

5G wireless communication systems and a series of perspectives on conceptualized 6G systems have emerged as powerful platforms for multiple use cases such as augmented/virtual reality (AR/VR), teleoperated surgery, and automatic driving [1,2]. An enhanced ultrareliable and low-latency communication (URLLC) has been proposed to support more stringent requirements on reliability and latency to meet the demands of those services. Specifically, the envisioned 6G wireless network may require URLLC based on a $10^{-10}$ packet loss probability and an over-the-air latency on the order of 0.1 ms [3,4].

With latency at a submillisecond level, short-packet transmissions are necessary [5]. Therefore, we should consider finite-blocklength channel coding to ensure reliability in URLLC. For the finite-blocklength (FBL) regime, the authors of [6] formulated the maximal coding rate of FBC over additive white Gaussian noise (AWGN) channels. The coding rate analysis has been extended to other practical scenarios, e.g., multiple-antenna fading channels [7], multiple-antenna Rayleigh-fading channels [8], block-fading channels [9], and hybrid automatic repeat request (HARQ) [10]. Latency is not only due to the physical layer transmission discussed in the literature above, but is also caused by the queuing of data packets in the network layer [11]. With a cross-layer design of variable-length coding for a single link, we have also achieved extremely low-latency communications in [12]. The cross-layer design for URLLC was also discussed in [13,14]. The author of [13] minimized the power consumption with a reliability constraint by adopting a packet-dropping policy. In [14], the violation probabilities of the maximal delay and peak-age of information were given for URLLC.

Nonterrestrial communications are also at the forefront of 6G technology research [15], in order to provide three-dimensional (3D) coverage by complementing on-the-ground infrastructures with aerial platforms, including satellites. For large-scale communication systems, the signal-to-noise ratio, which decays rapidly with distance, is a crucial factor that affects system performance. For this reason, relaying is essential to gain higher reliability. Relay communication began to attract researchers’ attention from the classical work of [16]. Various relaying schemes, including fixed relaying, selection relaying, and incremental relaying, were first discussed by Laneman, Tse, and Wornell in [17]. Different cooperative schemes between the direct link and the relay link were discussed under both amplify-and-forward (AF) or decode-and-forward (DF) modes in [18]. In [19], we extended the decode-and-cancel protocol proposed in our previous work [20] to cancel inter-relay interference (IRI) in the scheme CAO-SIR. In CAO-SIR, we achieved an equivalent parallel relay model for DF-based successive relaying since the interference is entirely canceled by delicately adjusting the transmission order of relay nodes.

Recently, researchers have also investigated relaying under the finite-blocklength regime (FBL). The authors in [21] derived closed-form expressions for the coding rates of relay communications under the Nakagami-m fading channel in the finite-blocklength regime. In [22], the researchers studied the relaying throughput performance of the quasistatic Rayleigh channels under the finite-blocklength regime, optimized the optimal distance and blocklength for URLLC, and compared that to the infinite-blocklength regime (IBL). However, relaying communications over a long distance suffer from technical difficulties such as the deterioration of the signal-to-noise ratio caused by the long distance, the increased latency caused by the increased number of relays, and the unclear theoretical upper limit of the optimal delay caused by the speed of light. In [21,22], large-scale communication was not taken into consideration, and the authors’ model was based on a fixed resource allocation and relay deployment scheme. Though [23] considered resource allocation for blocklength and power, this did not include a latency constraint because it was based on infinite-blocklength coding and a Poisson field of interferers. Thus, achieving low-latency short-packet communications over a large spatial scale is still an open problem.

In this paper, we focus on long-distance and short-packet communication. We aim to minimize the overall error probability while limiting the power cost under a time constraint. We prove the convexity of the overall error probability with respect to the blocklength and power under a particular condition when the relays are placed equidistantly. We also find a blocklength power allocating scheme to minimize the error probability, where more power should be allocated to the relays that have fewer encoded symbols. By scaling the problem conditions, we simplify the problem and obtain an analytical expression of the optimal error probability. Thereby, we find that the optimal relaying strategy is to allocate the blocklength and power equally to each relay. Then, we analyze the theoretical limits of the optimal solution. The discussion includes two cases, where the number of relays is fixed and the number of relays is variable. When there is a fixed number of relays in the communication system, we find that the error probability decreases superexponentially with the blocklength and the logarithm of the signal-to-noise ratio. When the number of relays is not fixed, we find there is an optimal number of relays in the relaying communication system. Therefore, the performance of relay communication has a theoretical upper limit. Under certain circumstances, no matter how many relays are added, the performance of the communication system cannot be improved. Based on a numerical simulation, we solve the optimization problem and compare it to our theoretical result.

The rest of this paper is organized as follows. Section 2 presents the system model. In Section 3, we form an optimization problem for the overall error probability in terms of the distance, blocklength, and signal-to-noise ratio. Then, we give a theoretical solution for the optimal bit-error rate. In Section 4, we analyze the theoretical asymptotic limit and approximate results of the optimal solution. In Section 5, we verify the correctness of our conclusions by a numerical simulation. Finally, conclusions and future suggestions are provided in Section 6.

## 2. System Model

**Figure 1:**
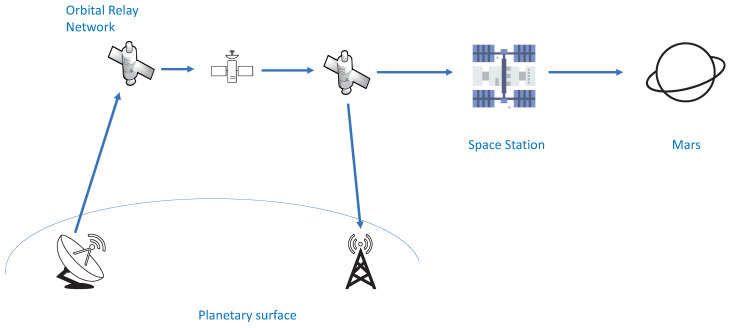
An orbital relaying system.

**Figure 2:**
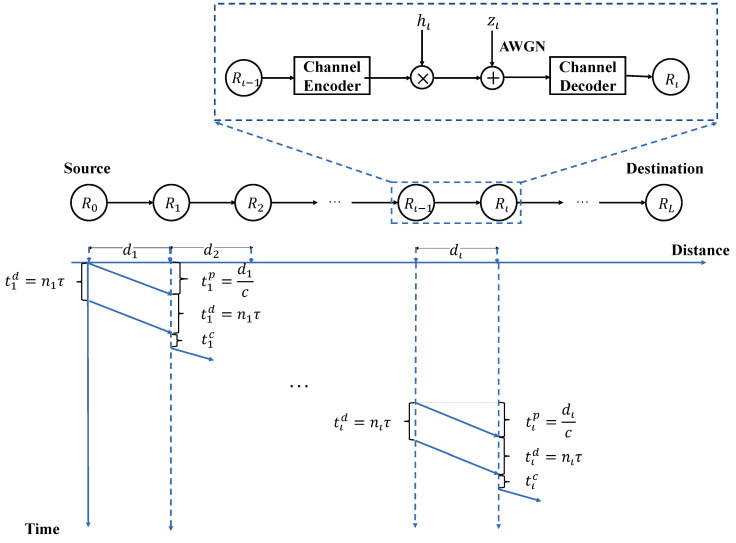
System model: a communication system with L−1 relays.

We focus on an orbital wireless system with the requirement of extremely low latency, which is demonstrated in Figure 1. The satellites communicate with another planet, e.g., Mars, via the space station. Each of the satellites is considered as a DF relay. For simplicity, we only consider the transmission of one packet from the source to destination, as shown in Figure 2, where a source node $R_{0}$ transmits a *k*-bit packet in each transmission to its destination node $R_{L}$ with the help of L−1 decode-and-forward (DF) relays, called $R_{1},R_{2},\cdots ,R_{L-1}$. The distance between node $R_{\iota -1}$ and $R_{\iota }$ is $d_{\iota }$. The *k*-bit packet is encoded into symbols to be transmitted from $R_{0}$ to $R_{L}$. The modulated waveforms of those symbols are reliably sent by node $R_{\iota -1}$. Node $R_{\iota }$ receives and detects it without distortion. We denote by τ the average time consumed in sending one symbol. As a result, $\frac{1}{\tau }$ is the symbol rate. Let $n_{\iota }$ be the number of symbols transferred between node $R_{\iota -1}$ and $R_{\iota }$, $n_{\iota }\in N_{+}$.

We assume node $R_{\iota -1}$ encodes the *k*-bit packet into $n_{\iota }$ symbols. $R_{\iota -1}$ sends a symbol vector $x_{\iota }$ to $R_{\iota }$. In particular, $x_{\iota }=\left(x_{\iota 1},x_{\iota 2},\cdots ,x_{\iota n_{\iota }}\right)$ and $|x_{\iota }{|}_{2}^{2}=1$. $x_{\iota j}$ is the *j*th symbol. Let $h_{\iota }$ denote the channel coefficient between node $R_{\iota -1}$ and $R_{\iota }$. Let $g_{\iota }$ denote the channel gain, namely $g_{\iota }={\left|h_{\iota }\right|}^{2}$. We denote by $y_{\iota }$ the received symbol vector at node $R_{\iota }$. Specifically, $y_{\iota }=\left(y_{\iota 1},y_{\iota 2},\cdots ,y_{\iota n_{\iota }}\right)$. For $1\leq j\leq n_{\iota }$, $y_{\iota j}$ can be presented by:

(1) $$y_{\iota j}=h_{\iota }x_{\iota j}+z_{\iota },$$

where $z_{\iota }$ is the additive Gaussian white noise. Let $d_{\iota }$ denote the distance between node $R_{\iota -1}$ and $R_{\iota }$. The path loss of the transmission from $R_{\iota -1}$ to $R_{\iota }$ is normalized as $d_{\iota }^{-\alpha }$, where α is the loss exponent. Due to the large scale of our model, the shadow-fading component can be ignored, and the channel gain is presented as $g_{\iota }=d_{\iota }^{-\alpha }$ [24]. We assume the signal-to-noise ratio (SNR) of the signal emitted by $R_{\iota -1}$ is $\gamma_{\iota }$. Thus, node $R_{\iota }$ receives the signal with the SNR equal to $\gamma_{\iota }d^{-\alpha }$ and sends it to the next node $R_{\iota +1}$ with the SNR equal to $\gamma_{\iota +1}$ if $R_{\iota }$ detects no error.

We then present the overall reliability from the source to the destination. To achieve extremely low latency and ultrareliability, a finite-blocklength coding scheme is adopted. For the real AWGN channel, a tight bound on the coding rate was given by [6]. Let $\epsilon_{\iota }$ denote the error probability from node $R_{\iota -1}$ to $R_{\iota }$. Since $R_{\iota -1}$ transmits *k* bits on $n_{\iota }$ symbols, the coding rate is $\frac{k}{n_{\iota }}$. Using the normal approximation in this paper, we have the formula for the channel coding rate in the transmission from $R_{\iota -1}$ and $R_{\iota }$, i.e.,

(2) $$\frac{k}{n_{\iota }}=C_{\iota }-\sqrt{\frac{V_{\iota }}{n_{\iota }}}Q^{-1}\left(\epsilon_{\iota }\right),$$

in which $Q\left(x\right)=\int_{x}^{\infty }\frac{1}{\sqrt{2\pi }}\exp\left(-\frac{t^{2}}{2}\right)dt$ is the Gaussian Q-function. $Q^{-1}$ is the inverse function of Q(x). We denote by $C_{\iota }$ and $V_{\iota }$ the channel capacity and the channel diversity, respectively. Under our configuration, the channel capacity is represented by:

(3) $$C_{\iota }=\log\left(1+\gamma_{\iota }d_{\iota }^{-\alpha }\right).$$

The channel dispersion is represented by:

(4) $$V_{\iota }=\frac{{\left(\gamma_{\iota }d_{\iota }^{-\alpha }+1\right)}^{2}}{\gamma_{\iota }d_{\iota }^{-\alpha }\left(\gamma_{\iota }d_{\iota }^{-\alpha }+2\right)}\log_{2}e.$$

Substituting Equation (3) into Equation (2), we present the error probability $\epsilon_{\iota }$:

(5) $$\epsilon_{\iota }=Q\left(\left(\ln\left(1+\gamma_{\iota }d_{\iota }^{-\alpha }\right)-\frac{k\ln2}{n_{\iota }}\right)\frac{n_{\iota }}{V_{\iota }}\right).$$

We assume all relay nodes and the destination node can decode the data packets and detect the error reliably. The relay nodes will forward the messages if and only if no error occurs. As a result, the overall error probability is given by:

(6) $$\begin{array}{cc} \epsilon & =1-\prod_{\iota =1}^{L}\left(1-\epsilon_{\iota }\right)\\ \; & \approx \sum_{\iota =1}^{L}\epsilon_{\iota },. \end{array}$$

The first-order approximation in Equation (6) gives us a reliable result when $\epsilon_{\iota }\approx 0$.

Then, we focus on the overall latency from the source to the destination. Since the data packet is transmitted serially, the overall latency *T* is the sum of the latency from $R_{\iota -1}$ to $R_{\iota }$, named $t_{\iota }$.

(7) $$T=\sum_{\iota =1}^{L}t_{\iota }.$$

In the URLLC communication without retransmission, the physical layer latency can be divided into the following components [11]:

(8) $$t_{\iota }=t_{\iota }^{c}+t_{\iota }^{p}+t_{\iota }^{d},$$

where $t_{\iota }^{c}$ is the time consumed by channel coding, $t_{\iota }^{p}$ is the time consumed by electronic wave propagation, and $t_{\iota }^{d}$ is the time consumed by decoding messages. Thus, we have:

(9) $$t_{\iota }^{d}=n_{\iota }\tau .$$

The propagation delay $t_{\iota }^{p}$ is caused by the signal travel time with the speed of light *c*, which is presented by:

(10) $$t_{\iota }^{p}=\frac{d_{\iota }}{c}.$$

We assume that each relay is equipped with a strong calculation force so that the time consumed by encoding messages is negligible for $t_{\iota }^{d}$ and $t_{\iota }^{p}$. Thus, the time to transfer for the data packet from $R_{\iota -1}$ to $R_{\iota }$ is:

(11) $$t_{\iota }=n_{\iota }\tau +\frac{d_{\iota }}{c}.$$

To meet the latency constraint, the overall delay $\sum_{i=1}^{L}t_{\iota }$ is no greater than a constant $T^{th}$, which gives:

(12) $$\sum_{\iota =1}^{L}n_{\iota }\tau +\sum_{\iota =1}^{L}\frac{d_{\iota }}{c}\leq T^{th}.$$

$T^{th}$ represents the maximal tolerable latency from the source to the destination. Since $d_{1},d_{2},\cdots ,d_{L}$ are constants when the positions of the relays are fixed, Equation (12) can be simplified as:

(13) $$\sum_{\iota =1}^{L}n_{\iota }\leq N^{th}\left(T^{th}\right),$$

where $N^{th}\left(T^{th}\right)$ is a linear function of $T^{th}$, i.e., $N^{th}\left(T^{th}\right)=\frac{T^{th}-\sum_{\iota =1}^{L}d_{\iota }}{\tau }$. By this means, we rewrite the latency constraint in Equation (12) as Equation (13), which is given as a constraint on the overall blocklength.

We consider an overall energy constraint assumption. The energy to transmit the packet from $R_{\iota -1}$ to $R_{\iota }$ equals $n_{\iota }\tau \gamma_{\iota }N_{0}$, for 1≤ι≤L. $N_{0}$ denotes the single-side noise density. Since τ and $N_{0}$ are constants, we have the normalized energy constraint:

(14) $$\sum_{\iota =1}^{L}n_{\iota }\gamma_{\iota }\leq E^{th},$$

where $E^{th}$ is the overall normalized energy of all source nodes and relay nodes.

The error probability $\epsilon_{\iota }$ is a function of blocklength $n_{\iota }$ and signal-to-noise ratio $\gamma_{\iota }$, as shown in Equation (5).

We next minimize the error probability ϵ subject to the energy and latency constraints.

## 3. Optimal Relay Deployment and Resource Allocation

In this section, we formulate the optimal tradeoff among the latency, normalized energy, and error probability. For this purpose, we first present the overall error probability in terms of the blocklength $n_{\iota }$ and signal-to-noise ratio $\gamma_{\iota }$ of each node $R_{\iota }$. Then, the optimization problem is formulated to minimize the error probability with constraints on the overall blocklength and normalized energy.

### 3.1. Problem Formulation

We let $Q\left(Y(\gamma_{\iota },d_{\iota },n_{\iota })\right)$ be the error probability of the link from $R_{\iota -1}$ to $R_{\iota }$. Y is given by:

(15) $$Y\left(\gamma ,d,n\right)=\left(\ln\left(1+\gamma d^{-\alpha }\right)-\frac{k\ln2}{n}\right)\sqrt{\frac{n{\left(\gamma d^{-\alpha }+1\right)}^{2}}{\gamma d^{-\alpha }\left(\gamma d^{-\alpha }+2\right)}}.$$

We denote by *D* the summation of $d_{\iota }$. Then, $\sum_{\iota =1}^{L}d_{\iota }=D$. In Section 2, we have the latency (blocklength) constraint in Equation (13) and the energy constraint in Equation (14). From the previous discussion, we establish the optimization problem (16) to minimize the error probability by allocating blocklength and power to each relay:

(16a) $$\begin{array}{cc} \min_{\gamma_{\iota },d_{\iota },n_{\iota }}\;\epsilon =\sum_{\iota =1}^{L} & Q\left(Y\left(\gamma_{\iota },d_{\iota },n_{\iota }\right)\right) \end{array}$$

(16b) $$\begin{array}{cc} s.t.\;\;\sum_{\iota =1}^{L}n_{\iota }\gamma_{\iota } & \leq E^{th}, \end{array}$$

(16c) $$\begin{array}{cc} \sum_{\iota =1}^{L}d_{\iota } & =D, \end{array}$$

(16d) $$\begin{array}{cc} \sum_{\iota =1}^{L}n_{\iota } & \leq N^{th}, \end{array}$$

(16e) $$\begin{array}{cc} \gamma_{\iota } & \geq 0, \end{array}$$

(16f) $$\begin{array}{cc} n_{\iota } & \in N_{+}. \end{array}$$

The optimization problem (16) is subject to the relay placement in Equation (16c) and the finite-blocklength coding in Equations (16b) and (16d). We consider a case where the blocklength of a finite-blocklength code tends to infinity, i.e., $N^{th}\to \infty$. As the overall blocklength $N^{th}$ increases, the effect of finite-blocklength coding is no longer apparent. There is no loss of generality in assuming the same coding process for each relay. Therefore, in this case, a uniform distribution of relays minimizes the error probability. Thus, when we consider the general FBC case, unless otherwise stated, we assume that the relays are deployed equidistantly. We then only consider resource allocation in the finite-blocklength coding. Furthermore, this deployment strategy can also be verified as the optimal one in the simulation results in Section 6.

Then, we simplify Problem (16) under the assumption that relays are placed equidistantly. The error probability ϵ given by (16a) is a decreasing function of $n_{\iota }$ and $\gamma_{\iota }$. Thus, we can certainly rewrite the inequations in Equations (16b) and (16d) as equations. Moreover, we expand the domain of $n_{\iota }$ to real numbers in the interval [1,∞) to avoid the complex integer programming.

In the case where relays are placed equidistantly, we denote by $Q\left(G(\gamma ,n)\right)$ the error probability to transfer *k* bits from $R_{\iota -1}$ to $R_{\iota }$, where:

(17) $$G\left(\gamma ,n\right)=\left(\ln\left(1+\gamma \right)-\frac{k\ln2}{n}\right)\sqrt{\frac{n{\left(\gamma +1\right)}^{2}}{\gamma \left(\gamma +2\right)}}.$$

With the discussion above, we have the optimization problem (18).

(18a) $$\begin{array}{cc} \min_{\gamma_{\iota },n_{\iota }}\;\epsilon =\sum_{\iota =1}^{L} & Q(G\left(\gamma_{\iota },n_{\iota }\right)) \end{array}$$

(18b) $$\begin{array}{cc} s.t.\;\;\sum_{\iota =1}^{L}n_{\iota }\gamma_{\iota } & \leq E^{th}, \end{array}$$

(18c) $$\begin{array}{cc} \sum_{\iota =1}^{L}n_{\iota } & \leq N^{th}, \end{array}$$

(18d) $$\begin{array}{cc} \gamma_{\iota } & \geq 0, \end{array}$$

(18e) $$\begin{array}{cc} n_{\iota } & \geq 1, \end{array}$$

The $\gamma_{\iota }$ in Problem (18) denotes the received SNR with a path loss of $d^{-\alpha }$ from $\gamma_{\iota }$ in Problem (16). This is the same situation for the constant $E^{th}$.

### 3.2. Properties of the Optimal Resource Allocation

Problem (18) is challenging due to its nonlinear constraint Equation (18b). Therefore, we relax the problem’s constraints. In particular, we present the following theorem.

**Theorem** **1.** *If $\ln\left(1+\gamma_{\iota }\right)-\frac{k\ln2}{n_{\iota }}>\max\left\{\frac{1}{k\ln2},\frac{1}{n_{\iota }}\right\}$, the ϵ in Problem (18) achieves its minimum when all the $\gamma_{\iota }$ are greater than a constant $\gamma_{0}$ and the sequence $n_{\iota }$ and $\gamma_{\iota }$ is in reverse order, i.e., for any $n_{i}\geq n_{j}$, we have $\gamma_{i}\leq \gamma_{j}$.*

**Proof** **of Theorem 1.** We use the notation φ(γ,n) to denote Q(G(γ,n)). For any *n* and any $\gamma_{1},\gamma_{2}$ satisfying the constraints of Problem (18) and $\gamma_{1}>\gamma_{2}$, let $\chi \left(n\right)=\varphi \left(n,\gamma_{2}\right)-\varphi \left(n,\gamma_{1}\right)$. We first prove that χ(n) is a decreasing function in its domain. We present the derivative of the function χ(n) as:

(19) $$\chi^{{}^{\prime }}\left(n\right)=\frac{1}{\sqrt{2\pi }}\left(-\exp\left(-\frac{G_{2}^{2}}{2}\right)\frac{\partial G_{2}}{\partial n}+\exp\left(-\frac{G_{1}^{2}}{2}\right)\frac{\partial G_{1}}{\partial n}\right).$$

We next show $\chi^{{}^{\prime }}\left(n\right)<0$, which is equivalent to the following inequation by substituting $G_{n}^{{}^{\prime }}$ and $G_{\gamma }^{{}^{\prime }}$ in Appendix A.

(20) $$\exp\left(\frac{G_{2}^{2}-G_{1}^{2}}{2}\right)<\frac{\ln\left(1+\gamma_{2}\right)+\frac{k\ln2}{n}}{\ln\left(1+\gamma_{1}\right)+\frac{k\ln2}{n}}\;\frac{\gamma_{2}+1}{\gamma_{1}+1}\;\sqrt{\frac{\gamma_{1}}{\gamma_{2}}\frac{\gamma_{1}+2}{\gamma_{2}+2}}.$$

Because $\gamma_{1}>\gamma_{2}$, we derive Equation (21) and Equation (22).

(21) $$\frac{\ln(1+\gamma_{2})}{\ln(1+\gamma_{1})}<\frac{\ln\left(1+\gamma_{2}\right)+\frac{k\ln2}{n}}{\ln\left(1+\gamma_{1}\right)+\frac{k\ln2}{n}},$$

(22) $$\frac{\gamma_{2}+1}{\gamma_{1}+1}\sqrt{\frac{\gamma_{1}}{\gamma_{2}}\frac{\gamma_{1}+2}{\gamma_{2}+2}}>1.$$

To prove Equation (20), we let $\zeta \left(\gamma \right)=\exp\left(-\frac{G^{2}\left(\gamma ,n\right)}{2}\right)\ln\left(1+\gamma \right)$. By calculating its derivative, we obtain:

(23) $$\zeta^{{}^{\prime }}\left(\gamma \right)=\exp\left(-\frac{G^{2}}{2}\right)\left(\frac{1}{1+\gamma }-GG_{\gamma }^{{}^{\prime }}\ln\left(1+\gamma \right)\right).$$

Because $\ln\left(1+\gamma \right)-\frac{k\ln2}{n}>\frac{1}{n}$, we have:

(24) $$\begin{array}{cc} GG_{\gamma }^{{}^{\prime }}\ln\left(1+\gamma \right) & >\left(\ln\left(1+\gamma \right)-\frac{k\ln2}{n}\right)n{\left(\frac{{\left(\gamma +1\right)}^{2}}{\gamma (\gamma +2)}\right)}^{\frac{1}{2}}\frac{\ln(1+\gamma )}{\sqrt{\gamma (\gamma +2)}}\\ & >\frac{\gamma +1}{\gamma (\gamma +2)}\ln\left(1+\gamma \right)\\ & >\frac{1}{\gamma +1}. \end{array}$$

The last step holds when $\gamma >\gamma_{0}$, where $\gamma_{0}$ is the solution of the function $\ln\left(1+\gamma \right)=\frac{\gamma (\gamma +2)}{{\left(\gamma +1\right)}^{2}}$ and $\gamma_{0}\approx 1.22$.

Now, we proved that ζ(γ) decreases when $\gamma >\gamma_{0}$. Thus, $\zeta \left(\gamma_{1}\right)<\zeta \left(\gamma_{2}\right)$, which leads to:

(25) $$\exp\left(\frac{G_{2}^{2}-G_{1}^{2}}{2}\right)<\frac{\ln(1+\gamma_{2})}{\ln(1+\gamma_{1})}.$$

Combining Equations (21), (22), and (25), we can assert that Equation (20) holds, which gives us $\chi^{{}^{\prime }}\left(n\right)<0$. Now, let $n_{1}>n_{2}$. We have $\chi \left(n_{1}\right)<\chi \left(n_{2}\right)$, i.e.,

(26) $$\varphi \left(n_{1},\gamma_{2}\right)+\varphi \left(n_{2},\gamma_{1}\right)<\varphi \left(n_{1},\gamma_{1}\right)+\varphi \left(n_{1},\gamma_{1}\right),$$

in which $\gamma_{1}>\gamma_{2}$. According to Equation (26), it is sufficient to show that when $n_{\iota }$ and $\gamma_{\iota }$ are in reverse order, the error probability is smaller, which completes the proof. □

We consider the condition $\ln\left(1+\gamma_{\iota }\right)-\frac{k\ln2}{n_{\iota }}>\max\left\{\frac{1}{k\ln2},\frac{1}{n_{\iota }}\right\}$ in Theorem 1 to ensure the error probability is within a reasonable range. Noticing Equation (6) holds when $\epsilon_{\iota }\approx 0$, we force channel capacity $C_{\iota }$ to be relatively larger than the coding rate $\frac{k}{n_{\iota }}$. If $C_{\iota }-\frac{k}{n_{\iota }}\approx 0$, we obtain $\epsilon_{\iota }\approx 0.5$ from Equation (5). For those $\epsilon_{\iota }$ being rather large, we can simply remove relay $R_{\iota }$, which does not affect the overall error probability. (Thus, we consider the condition $\ln\left(1+\gamma_{\iota }\right)-\frac{k\ln2}{n_{\iota }}>\max\left\{\frac{1}{k\ln2},\frac{1}{n_{\iota }}\right\}$ in the rest of the paper.)

By this means, we further shed some light on allocating the blocklength and power to the relays in Theorem 1. When the overall error probability is minimized, the blocklength allocated to a relay should be a decreasing function of its transmission power. According to Theorem 1, we present the following corollary.

**Corollary** **1.** *Problem (18) is equivalent to the following optimization problem:*

(27a) $$\begin{array}{cc} \min_{\gamma_{\iota },n_{\iota }}\;\epsilon =\sum_{\iota =1}^{L} & Q(G\left(\gamma_{\iota },n_{\iota }\right)) \end{array}$$

(27b) $$\begin{array}{cc} s.t.\;\;\sum_{\iota =1}^{L}n_{\iota }\gamma_{\iota } & =E^{th}, \end{array}$$

(27c) $$\begin{array}{cc} \sum_{\iota =1}^{L}n_{\iota } & =N^{th}, \end{array}$$

(27d) $$\begin{array}{cc} n_{1}\geq n_{2} & \geq \cdots \geq n_{L}\geq 1, \end{array}$$

(27e) $$\begin{array}{cc} 0\leq \gamma_{1} & \leq \gamma_{2}\leq \cdots \leq \gamma_{L}. \end{array}$$

**Proof** **of Corollary 1.** We start the proof by observing that each pair $\left(n_{\iota },\gamma_{\iota }\right)$ is independent in Problem (18) of the others. If we exchange the subscript of $\left(n_{i},\gamma_{i}\right)$ and $\left(n_{j},\gamma_{j}\right)$ for i≠j, the objective ϵ does not change. Thus, we can assume $n_{1}\geq n_{2}\geq \cdots \geq n_{L}$. Then, from Theorem 1, we know that Problem (18) achieves the minimum value when $\gamma_{1}\leq \gamma_{2}\leq \cdots \leq \gamma_{L}$.

We denote by $\epsilon_{1}^{*}$ and $\epsilon_{2}^{*}$ the optimal value of Problem (18) and Problem (27), respectively. Because the domain of Problem (27) is a part of the domain of Problem (18), $\epsilon_{1}^{*}\leq \epsilon_{2}^{*}$. Moreover, $\epsilon_{1}^{*}$ is achieved when $\gamma_{1}\leq \gamma_{2}\leq \cdots \leq \gamma_{L}$ from Theorem 1, which satisfies (27d) and (27e). Therefore, the optimal solution of Problem (18) falls into the domain of Problem (27). This gives $\epsilon_{2}^{*}\leq \epsilon_{1}^{*}$. Thus, $\epsilon_{1}^{*}=\epsilon_{2}^{*}$, which completes the proof. □

### 3.3. An Approximate, but Analytical Solution

Finally, we formulate an optimization problem with the overall SNR and blocklength constraints. To obtain an analytical solution to the optimal error probability, we first handle the nonlinear constraint in Equation (27b) by approximation. Then, we add a few conditions to simplify the proof.

We present the following lemma to approximate the overall power in Equation (29).

**Lemma** **1** (Rearrangement inequality [25])**.** *For $L\in N_{+}$ and two sequences satisfying $a_{1}\geq a_{2}\geq \cdots a_{L}$ and $b_{1}\geq b_{2}\geq \cdots b_{L}$,*

(28) $$\sum_{\iota =1}^{L}a_{\iota }b_{L+1-\iota }\leq \frac{1}{L}\left(\sum_{\iota =1}^{L}a_{\iota }\right)\left(\sum_{\iota =1}^{L}b_{\iota }\right)\leq \sum_{\iota =1}^{L}a_{\iota }b_{\iota }.$$

Equations (27b) and (27c) suggest that the sum of $\gamma_{\iota }$ can not exceed a certain value. Applying Lemma 1, we present the approximation for the overall SNR (Actually, the SNR is proportional to the transmission power in our model. Therefore, we also consider Equation (28) as the power constraint in the following text.) in Equation (29).

(29) $$\sum_{\iota =1}^{L}\gamma_{\iota }\approx \frac{LE^{th}}{N^{th}}.$$

Thus, we estimate the overall power by Equation (29). Finally, we obtain the optimization problem (30).

(30a) $$\begin{array}{cc} \min_{\gamma_{\iota },n_{\iota }}\;\epsilon =\sum_{\iota =1}^{L} & Q(G\left(\gamma_{\iota },n_{\iota }\right)) \end{array}$$

(30b) $$\begin{array}{cc} s.t.\;\;\sum_{\iota =1}^{L}\gamma_{\iota } & =\frac{LE^{th}}{N^{th}}, \end{array}$$

(30c) $$\begin{array}{cc} \sum_{\iota =1}^{L}n_{\iota } & =N^{th}, \end{array}$$

(30d) $$\begin{array}{cc} \max\left\{\frac{1}{k\ln2},\frac{1}{n_{\iota }}\right\} & <\ln\left(1+\gamma_{\iota }\right)-\frac{k\ln2}{n_{\iota }}, \end{array}$$

(30e) $$\begin{array}{cc} n_{1}\geq n_{2} & \geq \cdots \geq n_{L}\geq 0, \end{array}$$

(30f) $$\begin{array}{cc} 0\leq \gamma_{1} & \leq \gamma_{2}\leq \cdots \leq \gamma_{L}. \end{array}$$

In order to solve Problem (30), we have Theorem 2 for the convexity of Q(G(γ,n)).

**Theorem** **2.** *The function Q(G(γ,n)) is a binary convex function for the pair (γ,n) in the domain of Problem (30).*

**Proof** **of Theorem 2.** For a function f(x,y), we use the abbreviations $f_{x}^{{}^{\prime }}=\frac{\partial f}{\partial x}$, $f_{y}^{{}^{\prime }}=\frac{\partial f}{\partial y}$, $f_{xy}^{{}^{\prime \prime }}=\frac{\partial^{2}f}{\partial x\partial y}$, $f_{x}^{{}^{\prime \prime }}=\frac{\partial^{2}f}{\partial x^{2}}$, and $f_{y}^{{}^{\prime \prime }}=\frac{\partial^{2}f}{\partial y^{2}}$.

We focus on the Hessian matrix of the function H=Q(G(γ,n)), which is given by $\nabla^{2}H=\left[\begin{array}{cc} H_{\gamma }^{{}^{\prime \prime }} & H_{\gamma n}^{{}^{\prime \prime }}\\ H_{n\gamma }^{{}^{\prime \prime }} & H_{n}^{{}^{\prime \prime }} \end{array}\right]$. The main idea of the proof is to show that the Hessian matrix is positive definite. Actually, we calculate the partial derivatives of H as follows:

(31) $$H_{n}^{{}^{\prime \prime }}=\left(G{\left(G_{n}^{{}^{\prime }}\right)}^{2}-G_{n}^{{}^{\prime \prime }}\right)\exp\left(-\frac{G^{2}}{2}\right).$$

Similarly, we obtain:

(32) $$H_{\gamma }^{{}^{\prime \prime }}=\left(G{\left(G_{\gamma }^{{}^{\prime }}\right)}^{2}-G_{\gamma }^{{}^{\prime \prime }}\right)\exp\left(-\frac{G^{2}}{2}\right),$$

and:

(33) $$H_{n\gamma }^{{}^{\prime \prime }}=H_{\gamma n}^{{}^{\prime \prime }}=\left(GG_{n}^{{}^{\prime }}G_{\gamma }^{{}^{\prime }}-G_{n\gamma }^{{}^{\prime \prime }}\right)\exp\left(-\frac{G^{2}}{2}\right).$$

From Appendix A, we have $G_{n}^{{}^{\prime \prime }}<0$ and $G_{\gamma }^{{}^{\prime \prime }}<0$. Substituting the negative condition property into Equation (32), we have:

(34) $$\begin{array}{cc} H_{\gamma }^{{}^{\prime \prime }} & =\left({\left(G_{\gamma }^{{}^{\prime }}\right)}^{2}G-G_{\gamma }^{{}^{\prime \prime }}\right)\exp\left(-\frac{G^{2}}{2}\right)\\ & >{\left(G_{\gamma }^{{}^{\prime }}\right)}^{2}G\exp\left(-\frac{G^{2}}{2}\right)\\ & >0. \end{array}$$

Similarly, $H_{n}^{{}^{\prime \prime }}>0$.

To deal with the cross partial derivative $H_{n\gamma }^{{}^{\prime \prime }}$, we calculate the factor in the first round brackets in Equation (33) as:

(35) $$\begin{array}{cc} GG_{n}^{{}^{\prime }}G_{\gamma }^{{}^{\prime }}-G_{n\gamma }^{{}^{\prime \prime }}= & n^{-\frac{1}{2}}{\left[\gamma (\gamma +2)\right]}^{-\frac{3}{2}}\{\left[\frac{\gamma \left(\gamma +2\right)-\left(\ln\left(1+\gamma \right)-\frac{k\ln2}{n}\right)}{2}\right]\times \\ \; & \left[n\left(\ln\left(1+\gamma \right)-\frac{k\ln2}{n}\right)\left(\ln\left(1+\gamma \right)+\frac{k\ln2}{n}\right)-1\right]-\frac{k\ln2}{n}\}. \end{array}$$

Since $\ln\left(1+\gamma \right)>\frac{k\ln2}{n}$, we have:

(36) $$\begin{array}{cc} \left(n\left(\ln\left(1+\gamma \right)-\frac{k\ln2}{n}\right)\left(\ln\left(1+\gamma \right)+\frac{k\ln2}{n}\right)-1\right) & >\left(2n\left(\ln\left(1+\gamma \right)-\frac{k\ln2}{n}\right)\frac{k\ln2}{n}-1\right)\\ & =2k\ln2\left(\ln\left(1+\gamma \right)-\frac{k\ln2}{n}\right)-1\\ & >1, \end{array}$$

which applies the condition of (30d). Thus,

(37) $$\begin{array}{cc} GG_{n}^{{}^{\prime }}G_{\gamma }^{{}^{\prime }}-G_{n\gamma }^{{}^{\prime \prime }}> & n^{-\frac{1}{2}}{\left[\gamma (\gamma +2)\right]}^{-\frac{3}{2}}\left[\frac{\gamma \left(\gamma +2\right)-\ln\left(1+\gamma \right)-\frac{k\ln2}{n}}{2}\right]\\ > & n^{-\frac{1}{2}}{\left(\gamma (\gamma +2)\right)}^{-\frac{3}{2}}\left[\frac{\gamma (\gamma +2)-2\ln(1+\gamma )}{2}\right]\\ > & 0. \end{array}$$

Since $GG_{n}^{{}^{\prime }}G_{\gamma }^{{}^{\prime }}-G_{n\gamma }^{{}^{\prime \prime }}>0$ and $G_{n\gamma }^{{}^{\prime \prime }}<0$, from Appendix A, we obtain:

(38) $$\begin{array}{cc} H_{n\gamma }^{{}^{\prime \prime }}H_{\gamma n}^{{}^{\prime \prime }} & ={\left(GG_{n}^{{}^{\prime }}G_{\gamma }^{{}^{\prime }}-G_{n\gamma }^{{}^{\prime \prime }}\right)}^{2}\exp\left(-G^{2}\right)\\ \; & <{\left(GG_{n}^{{}^{\prime }}G_{\gamma }^{{}^{\prime }}\right)}^{2}\exp\left(-G^{2}\right)\\ & <H_{n}^{{}^{\prime \prime }}H_{\gamma }^{{}^{\prime \prime }}. \end{array}$$

This yields that $\nabla^{2}H$ is positive definite. The proof is complete. □

Theorem 2 proves the convexity of the objective function mathematically. Furthermore, we have the optimal solution of Problem (30) as follows.

**Theorem** **3.** *The optimal solution of Problem (30) is $LQ\left(G\left(\frac{E^{th}}{N^{th}},\frac{N^{th}}{L}\right)\right)$, when we set $n_{\iota }=\frac{N^{th}}{L}$ and $\gamma_{\iota }=\frac{E^{th}}{N^{th}}$ for each ι=1,⋯,L.*

**Proof** **of Theorem 3.** From Theorem 2, we have that Q(G(γ,n)) is a convex function. Applying Jensen’s inequality, we have:

(39) $$\epsilon \geq LQ\left(G\left(\frac{\sum_{\iota =1}^{L}\gamma_{\iota }}{L},\frac{\sum_{\iota =1}^{L}n_{\iota }}{L}\right)\right)=LQ\left(G\left(\frac{E^{th}}{N^{th}},\frac{N^{th}}{L}\right)\right).$$

The proof is complete. □

Theorem 3 states that the system achieves the highest reliability when the transmission power and blocklength of the relays are the same. Furthermore, we obtain an analytical expression of the optimal error probability. This relay resource allocation strategy is intuitively reasonable, corresponding to the equal distance between each successive relay pair. Finally, from the discussion above, we generate a suboptimal result of the initial problem.

## 4. Asymptotic Performance Analysis for Long-Distance Short-Packet Transmission

In this section, we focus on the solution given by Theorem 3, i.e., $\epsilon =LQ\left(G\left(\frac{E^{th}}{N^{th}},\frac{N^{th}}{L}\right)\right)$. Let $\bar{\gamma }$ and $\bar{n}$ denote $\frac{E^{th}}{N^{th}}$ and $\frac{N^{th}}{L}$, respectively. To give more insight, we develop an asymptotic analysis of the error probability for large $\bar{n}$ or $\bar{\gamma }$.

To start with, we present Theorem 4 to approximate the overall error probability ϵ when $G\left(\frac{E^{th}}{N^{th}},\frac{N^{th}}{L}\right)\to \infty$.

**Theorem** **4.** *If $E^{th}$ or $N^{th}$ is large enough, the overall error probability is approximated by:*

(40) $$\epsilon \approx \frac{L^{\frac{3}{2}}\sqrt{\frac{E^{th}}{N^{th}}\left(\frac{E^{th}}{N^{th}}+2\right)}\exp\left(-\frac{N^{th}{\left(\ln\left(1+\frac{E^{th}}{N^{th}}\right)-\frac{Lk\ln2}{N^{th}}\right)}^{2}{\left(1+\frac{E^{th}}{N^{th}}\right)}^{2}}{2L\frac{E^{th}}{N^{th}}\left(\frac{E^{th}}{N^{th}}+2\right)}\right)}{\sqrt{2\pi N^{th}}\left(\ln\left(1+\frac{E^{th}}{N^{th}}\right)-\frac{Lk\ln2}{N^{th}}\right)\left(1+\frac{E^{th}}{N^{th}}\right)}.$$

**Proof.**  We notice that for any x>0, the following inequation holds:

(41) $$\frac{x}{1+x^{2}}\exp\left(-\frac{x^{2}}{2}\right)<\int_{x}^{+\infty }\exp\left(-\frac{u^{2}}{2}\right)du<\frac{1}{x}\exp\left(-\frac{x^{2}}{2}\right).$$

Substitute $x=G\left(\frac{E^{th}}{N^{th}},\frac{N^{th}}{L}\right)$ into Equation (41), and the proof is completed. □

From Theorem 4, we also express ϵ with $\bar{\gamma }$ and $\bar{n}$ as:

(42) $$\epsilon \approx \frac{L\sqrt{\bar{\gamma }\left(\bar{\gamma }+2\right)}\exp\left(-\frac{\bar{n}{\left(\ln\left(1+\bar{\gamma }\right)-\frac{k\ln2}{\bar{n}}\right)}^{2}{\left(1+\bar{\gamma }\right)}^{2}}{2\bar{\gamma }\left(\bar{\gamma }+2\right)}\right)}{\sqrt{2\pi \bar{n}}\left(\ln\left(1+\bar{\gamma }\right)-\frac{k\ln2}{\bar{n}}\right)\left(1+\bar{\gamma }\right)},$$

in which we recall that $\bar{n}=\frac{N^{th}}{L}$ and $\bar{\gamma }=\frac{E^{th}}{N^{th}}$.

### 4.1. Asymptotic Analysis with a Fixed Number of Relays

In this part, we assume that the number of relays is fixed as a constant *L*. When $\bar{n}$ or $\bar{\gamma }$ is large, we have $\left(\ln\left(1+\bar{\gamma }\right)-\frac{k\ln2}{n}\right)\approx (\ln\left(1+\bar{\gamma }\right)$ and $\frac{{\left(1+\bar{\gamma }\right)}^{2}}{\bar{\gamma }\left(\bar{\gamma }+1\right)}\approx 1$. Thus, we approximate ϵ in Equation (43) by applying Theorem 4.

(43) $$\epsilon \approx \frac{L\exp\left(-\frac{\bar{n}\ln^{2}\left(1+\bar{\gamma }\right)}{2}\right)}{\sqrt{2\pi \bar{n}}\ln\left(1+\bar{\gamma }\right)}.$$

For a large $\bar{n}$, we present ϵ as a function of $\bar{n}$, i.e.,

(44) $$\epsilon \approx \frac{\mu_{1}\exp\left(-\theta_{1}n\right)}{\sqrt{n}},$$

where $\mu_{1}=\frac{L}{\sqrt{2\pi }\ln\left(1+\bar{\gamma }\right)}$ and $\theta_{1}=-\frac{\ln^{2}\left(1+\bar{\gamma }\right)}{2}$. In this case, as:

(45) $$\begin{array}{cc} \bar{n} & =\frac{N^{th}}{L}\\ & =\frac{T-\frac{D}{c}}{L\tau }\to \infty , \end{array}$$

the whole problem degenerates into an infinite-blocklength problem. The well-known Shannon formula can be applied to assure ϵ→0 with a given data rate. Moreover, from (44), we can see that ϵ decreases nearly exponentially with *n*.

For a large $\bar{\gamma }$, we present ϵ as a function of $\bar{\gamma }$, i.e.,

(46) $$\epsilon \approx \frac{\mu_{2}{\bar{\gamma }}^{-\theta_{2}\ln\left(\bar{\gamma }\right)}}{\ln(\bar{\gamma })},$$

where $\mu_{2}=\frac{L}{\sqrt{2\pi \bar{n}}}$ and $\theta_{2}=-\frac{\bar{n}}{2}$. From Equation (46), we can see that ϵ decreases nearly exponentially with $\log(\bar{\gamma })$, which is an infinitesimal of higher order compared to any fractional polynomials, but a low-order infinitesimal compared to $\exp\left(-\bar{\gamma }\right)$.

### 4.2. Analysis of Relay Gains

In this part, we express error probability ϵ as a function of the number of relays *L*. In this case, the maximal normalized energy $E^{th}$ and the overall blocklength $N^{th}$ are a function of *L*. For instance, in practice, a longer distance means a more severe path loss, and in those cases, we should allocate more energy. We denote by $E^{th}\left(L\right)$ the maximal normalized energy and by $N^{th}\left(L\right)$ the overall blocklength, respectively. Certain types of $E^{th}\left(L\right)$ and $N^{th}\left(L\right)$ are now discussed. Define $E=E^{th}\left(1\right)$ and $N=N^{th}\left(1\right)$. We still use the same assumption in the last part to approximate ϵ.

First, we let $N^{th}\left(L\right)=N$ and $E^{th}\left(L\right)=E$. The constraints do not change regardless of the number of relays. From Equation (40), we express ϵ in terms of the number of relays *L*, i.e.,

(47) $$\epsilon \approx \mu_{3}L^{\frac{3}{2}}\exp\left(-\frac{\theta_{3}}{L}\right),$$

where $\mu_{3}=\frac{1}{\sqrt{2\pi N}\ln\left(1+\frac{E}{N}\right)}$ and $\theta_{3}=\frac{N\ln\left(1+\frac{E}{N}\right)}{2}$.

From Equation (47), the error probability increases with *L*. This is because both communication resources, i.e., the blocklength and transmission power, are fixed. The resources allocated to each relay decreases with *L*. Thus, the error probability of each hop increases with *L*.

Next, we let $E^{th}\left(L\right)$ increase linearly with *L*, i.e., $E^{th}\left(L\right)=LE$. Moreover, we let $N^{th}\left(L\right)=N$ be a fixed value. Under this assumption, we have:

(48) $$\epsilon \approx \frac{L^{\frac{3}{2}}\exp\left(-N\frac{\ln^{2}\left(1+\frac{LE}{N}\right)}{2L}\right)}{\sqrt{2\pi N}\ln\left(1+\frac{LE}{N}\right)}.$$

We denote by ϵ=F(L) the function of ϵ and *L* in Equation (48). By solving $F^{{}^{\prime }}\left(L\right)=0$, there is a local minimum point $L_{0}$. (Actually, $L_{0}$ may not be an integer. However, we do not use this fact in any essential way.) $F\left(L\right)\geq F\left(L_{0}\right)$ holds for all *L*. Thus, there are an optimal number of relays $L=L_{0}$ to minimize the error probability ϵ. When the number of relays *L* increases substantially, the error probability goes to infinity because there is a limited blocklength to be allocated. The discussion enlightens us that there is an optimal number of relays to choose in practice.

Then, we let $E^{th}\left(L\right)=LE$ and $N^{th}\left(L\right)=LN$. In this case, we assume that the maximal tolerable latency is proportional to *L*. It is a practical scenario when the total distance increases linearly with *L*. Under this consumption, we have Equation (49), i.e.,

(49) $$\epsilon \approx \frac{L\exp\left(-N\frac{\ln^{2}\left(1+\frac{E}{N}\right)}{2}\right)}{\sqrt{2\pi N}\ln\left(1+\frac{E}{N}\right)},$$

In this case, ϵ increases linearly with *L*.

Last but not least, we let $E^{th}\left(L\right)=L^{\alpha }E$ and $N^{th}\left(L\right)=N$. In this case, we increase the number of relays in a fixed total distance *D* from the source to the destination. The distance between each node is given by $\frac{D}{L}$. Thus, the received SNR of each relay is proportional to $L^{\alpha }$, i.e., $E^{th}\left(L\right)=L^{\alpha }E$. The error probability is given by:

(50) $$\epsilon \approx \frac{L^{\frac{3}{2}}\exp\left(-N\frac{\ln^{2}\left(1+\frac{L^{\alpha }E}{N}\right)}{2L}\right)}{\sqrt{2\pi N}\ln\left(1+\frac{L^{\alpha }E}{N}\right)}.$$

In Equation (50), there is also an optimal *L* to minimize ϵ.

To summarize, we investigate four cases in this part as *L* changes in Equations (47)–(50). More generally, we let $E^{th}\left(L\right),N^{th}\left(L\right)$ be any function of *L*. We have Equation (51) for the general case.

(51) $$\epsilon \approx \frac{L^{\frac{3}{2}}\sqrt{\frac{E^{th}\left(L\right)}{N^{th}\left(L\right)}\left(\frac{E^{th}\left(L\right)}{N^{th}\left(L\right)}+2\right)}\exp\left(-\frac{N^{th}\left(L\right){\left(\ln\left(1+\frac{E^{th}\left(L\right)}{N^{th}\left(L\right)}\right)-\frac{Lk\ln2}{N^{th}\left(L\right)}\right)}^{2}{\left(1+\frac{E^{th}\left(L\right)}{N^{th}\left(L\right)}\right)}^{2}}{2L\frac{E^{th}\left(L\right)}{N^{th}\left(L\right)}\left(\frac{E^{th}\left(L\right)}{N^{th}\left(L\right)}+2\right)}\right)}{\sqrt{2\pi N^{th}\left(L\right)}\left(\ln\left(1+\frac{E^{th}\left(L\right)}{N^{th}\left(L\right)}\right)-\frac{Lk\ln2}{N^{th}\left(L\right)}\right)\left(1+\frac{E^{th}\left(L\right)}{N^{th}\left(L\right)}\right)}.$$

### 4.3. The Blocklength–Power Tradeoff

In this part, we determine the blocklength and power tradeoff with a given maximal error probability $\epsilon^{\max}$.

Applying Theorem 3 and $\frac{{\left(\bar{\gamma }+1\right)}^{2}}{\bar{\gamma }\left(\bar{\gamma }+2\right)}\approx 1$, we have:

(52) $$Q^{-1}\left(\frac{\epsilon^{\max}}{L}\right)\approx \sqrt{\bar{n}}\left(\ln\left(1+\bar{\gamma }\right)-\frac{k\ln2}{\bar{n}}\right).$$

When the number of relays *L* is fixed, we can see that the average blocklength $\bar{n}$ should decrease with the average transmission power $\bar{\gamma }$. This result corresponds to the resource allocation property in Theorem 1.

When the number of relays *L* is not fixed, we can substitute the functions $E^{th}\left(L\right)$ and $N^{th}\left(L\right)$ into Equation (52). For example, since:

(53) $$\begin{array}{cc} \bar{\gamma } & =\frac{E^{th}\left(L\right)}{N^{th}\left(L\right)}\\ & =\frac{E^{th}\left(L\right)}{L\bar{n}}, \end{array}$$

we have:

(54) $$Q^{-1}\left(\frac{\epsilon^{\max}}{L}\right)\approx \sqrt{\bar{n}}\left(\ln\left(1+\frac{E^{th}\left(L\right)}{L\bar{n}}\right)-\frac{k\ln2}{\bar{n}}\right).$$

Similarly, we have:

(55) $$Q^{-1}\left(\frac{\epsilon^{\max}}{L}\right)\approx \sqrt{\frac{E^{th}\left(L\right)}{L\bar{\gamma }}}\left(\ln\left(1+\bar{\gamma }\right)-\frac{Lk\bar{\gamma }\ln2}{E^{th}\left(L\right)}\right).$$

Equations (54) and (55) indicate a tradeoff between $\bar{n}$ and $\bar{\gamma }$. Other types of $E^{th}\left(L\right)$ and $N^{th}\left(L\right)$ are also applicable. However, this topic exceeds the scope of this paper.

More importantly, it is worth pointing out that Equation (52) reveals whether a transmission is possible by increasing the number of relays with the given error probability, overall energy, and latency requirements.

## 5. Simulation Results

In this section, numerical and simulation results are presented to validate the theoretical analysis and demonstrate the advantages of our work.

**Figure 3:**
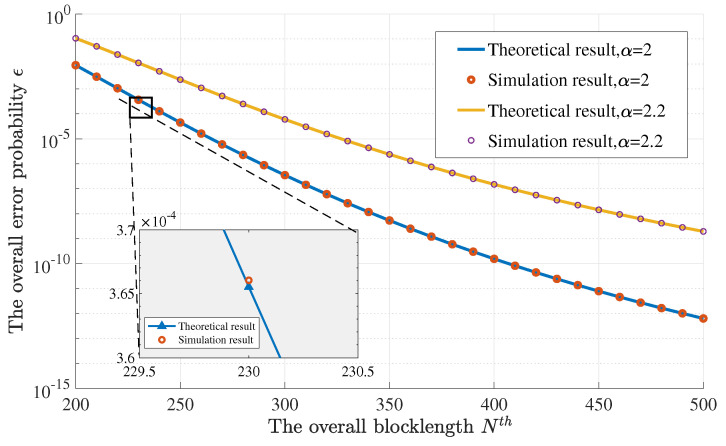
The numerical result of the optimal error probability–blocklength curve.

**Figure 4:**
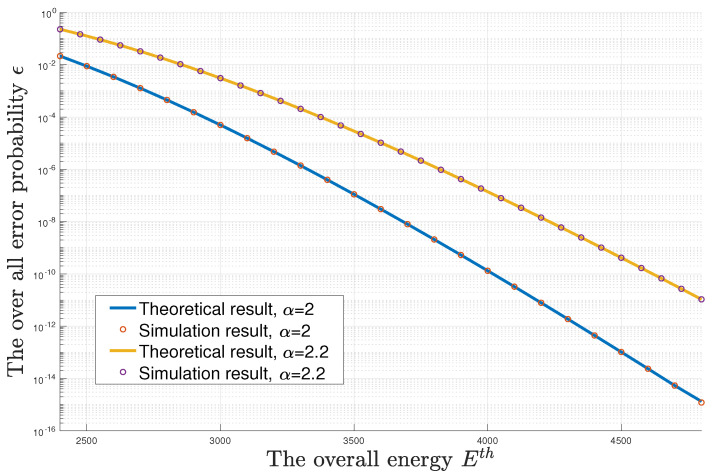
The numerical result of the optimal error probability–energy curve.

First, Figure 3 and Figure 4 present the error probability with respect to the overall blocklength and the overall energy, respectively. The two curves are drawn for the loss exponent α=2 and α=2.2. We consider the transmission of a one-hundred-bit data packet with the help of two relays, i.e., L=3. The distance from the source to the destination is D=6. The normalized unit distance is $d_{0}=1$. In Figure 3, the overall normalized energy $E^{th}$ is set as 4200 and the overall blocklength $N^{th}$ ranges from 200 to 500. In Figure 4, the overall blocklength $N^{th}$ is set as 450 and the overall normalized energy $E^{th}$ ranges from 2400 to 4800. In the subfigure of Figure 3, the relative error is less than $10^{-5}$, which is mainly caused by the rounding function of $\bar{n}=\left[\frac{N^{th}}{L}\right]$. In Figure 4, the relative error is less than $10^{-4}$, which is mainly caused by the approximation of ϵ in Equation (6). It is easy to see that the theoretical results obtained via Theorem 3 match well with their corresponding simulation results. Furthermore, we can see that a larger α leads to a higher error probability.

**Figure 5:**
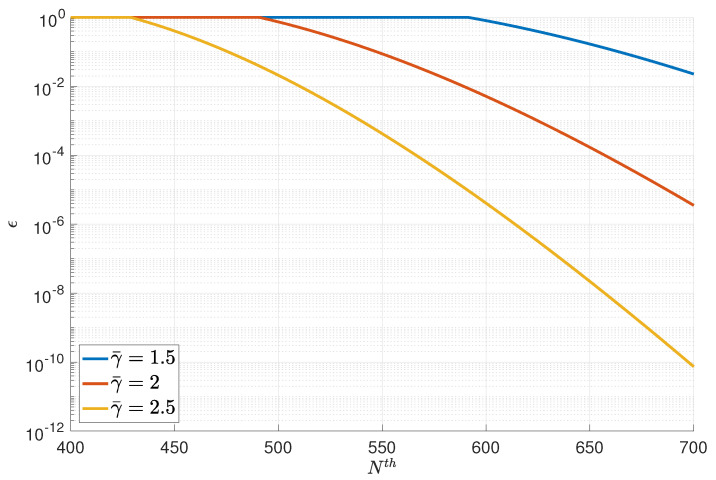
The overall blocklength versus error probability curves.

**Figure 6:**
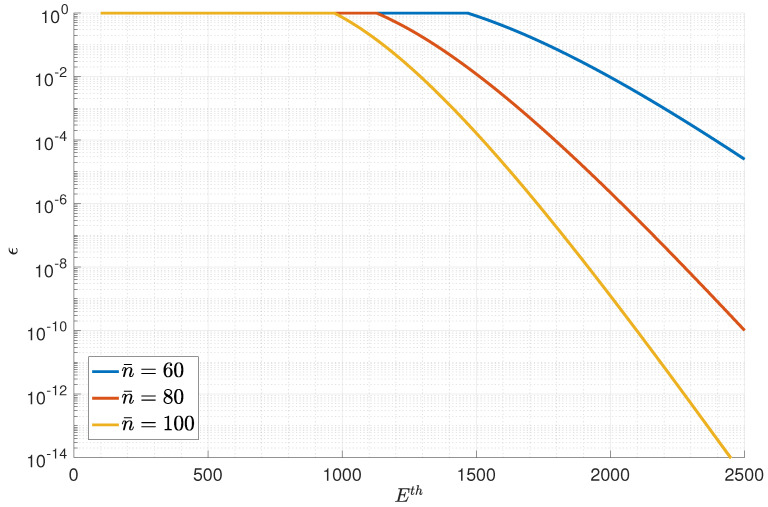
The overall normalized energy versus error probability curves.

Next, we present the numerical result of the optimal solution in Figure 5 and Figure 6. The number of relays was set as L=5. Figure 5 presents the overall blocklength versus the error probability curves for three average transmission powers, namely $\bar{\gamma }=1.5$, $\bar{\gamma }=2$, and $\bar{\gamma }=2.5$. Figure 6 presents the overall normalized energy versus error probability curves for three average blocklengths, namely $\bar{n}=60$, $\bar{n}=80$, and $\bar{n}=100$.

**Figure 7:**
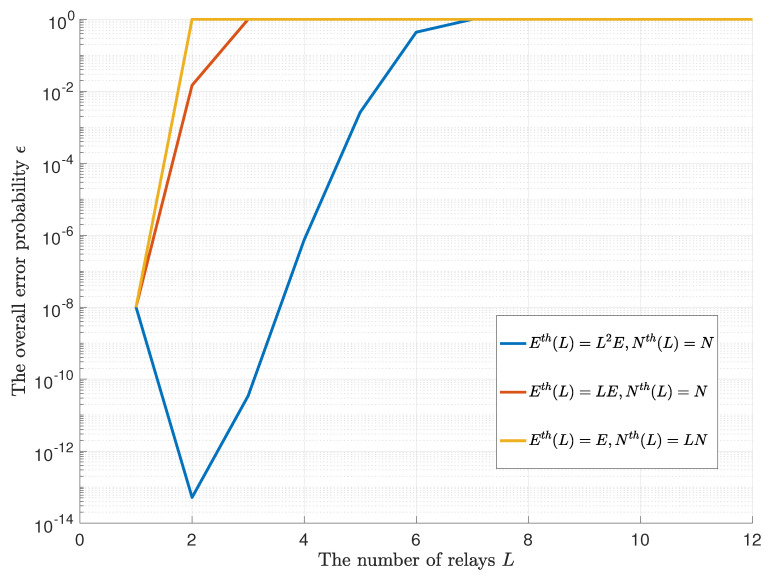
The optimal ϵ curves w.r.t the number of relays *L*.

In Figure 7, we present the optimal ϵ curves with respect to the number of relays *L* when different kinds of $E^{th}\left(L\right)$ and $N^{th}\left(L\right)$ are given. In most cases, the error probability increases with the number of relays *L*. However, under the circumstance where $E^{th}\left(L\right)=L^{2}E,N^{th}\left(L\right)=N$, we have an optimal L=2.

**Figure 8:**
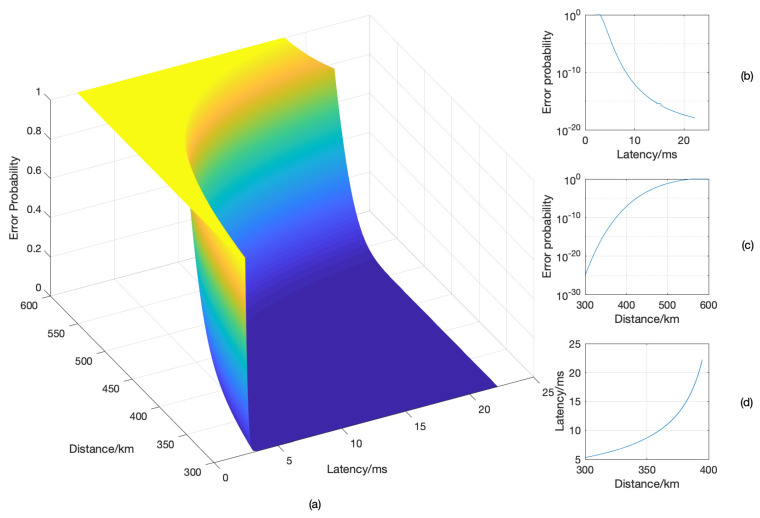
The optimal distance–latency–reliability tradeoff.

Finally, Figure 8 presents the optimal distance–latency–reliability tradeoff. The parameters were set as follows: the number of relays L=3, the overall normalized energy $E^{th}=4150$, and the symbol duration $\tau =2\times 10^{-4}$.

We chose the total distance D∈ [300 km,600 km]. The normalized distance was unitized by a factor cτ, where *c* is the speed of light. Figure 8a shows the reliability with respect to the distance and latency. Figure 8b–d presents the projection of the surface when the distance, latency, or reliability is fixed. Specifically, Figure 8b shows the reliability–latency tradeoff when the total distance is fixed as 360 km. Figure 8c presents the reliability–distance tradeoff when the latency is set as a constant of 10.14 ms. Figure 8d shows the latency–distance tradeoff when the error probability is $1\times 10^{-12}$.

From the simulation results, we can draw some conclusions about the advantages of our model as follows. First, we consider both propagation delay and decoding delay. Our model was established to assess the performance of large-scale low-latency communication systems, which has not been included in any other previous works. We obtained a low-complexity solution for the error probability by giving an analytical solution of the relaxed problem. Furthermore, we attained the best resource allocation policy. Last but not least, we determined the theoretical limit of our model, under the condition of whether the number of relays is fixed or not. In particular, our result determines the minimum error probability of relaying communication given the latency, power allocation scheme, and the relay nodes’ positions.

## 6. Conclusions

In this paper, we studied the optimal policies and performance bounds for low-latency short-packet transmission over a large spatial scale, in which both the propagation loss of the SNR and the propagation delay should be taken into consideration. Although DF relays can be deployed to regenerate packets reliably and mitigate their SNR loss due to propagation, they induce a severe decoding delay. To address these issues, we optimized the relay deployment, power allocation, and blocklength for each relay. Moreover, we investigated the performance limits of the distant transmission of short packets via asymptotic analysis. Given the overall transmission power, we found the optimal distance–latency–reliability tradeoff. Our results may provide some engineering insights into the design of integrated LEO satellite communications in 6G. Important future work includes the study of amplify-and-forward short-packet relaying, multi-user short-packet transmission, and two-way short-packet relaying over a large spatial scale. Furthermore, we will consider the situation where the relay nodes are not at fixed positions. We will investigate whether it is possible to allocate the resources in real time based on the positions of the nodes.

## Data Availability

Data is contained within the article or supplementary material.

## Appendix A. Derivatives of Function G

The partial derivatives of G(n,γ) are listed in this Appendix. Furthermore, we prove whether each of them is positive or negative.

The partial derivative of G w.r.t. *n* is:

(A1) $$\begin{array}{cc} G_{n}^{{}^{\prime }} & =\sqrt{\frac{{\left(\gamma +1\right)}^{2}}{\gamma (\gamma +2)}}\left(\frac{k\ln2}{2n^{\frac{3}{2}}}+\frac{\ln(1+\gamma )}{2n^{\frac{1}{2}}}\right)\\ & >0, \end{array}$$

and the partial derivative of G w.r.t. γ is:

(A2) $$\begin{array}{cc} G_{\gamma }^{{}^{\prime }} & =\sqrt{n}{\left[\gamma (\gamma +2)\right]}^{-\frac{3}{2}}\left[\gamma \left(\gamma +2\right)-\left(\ln\left(1+\gamma \right)-\frac{k\ln2}{n}\right)\right]\\ & >0. \end{array}$$

This is because $\gamma \left(\gamma +2\right)-\left(\ln\left(1+\gamma \right)-\frac{k\ln2}{n}\right)>\gamma -\ln\left(1+\gamma \right)>0$.

The second-order partial derivative of G w.r.t. *n* is given by:

(A3) $$\begin{array}{cc} G_{n}^{{}^{\prime \prime }} & =-\sqrt{\frac{{\left(\gamma +1\right)}^{2}}{\gamma (\gamma +2)}}\left(\frac{3k\ln2}{4n^{\frac{5}{2}}}+\frac{\ln(1+\gamma )}{4n^{\frac{3}{2}}}\right)\\ & <0. \end{array}$$

The second-order partial derivative of G w.r.t. γ is given by:

(A4) $$\begin{array}{cc} G_{\gamma }^{{}^{\prime \prime }}= & -\sqrt{n}{\left[\gamma (\gamma +2)\right]}^{-\frac{3}{2}}\left[\gamma +1+\frac{1}{\gamma +1}-\frac{3\left(\ln\left(1+\gamma \right)-\frac{k\ln2}{n}\right)\left(\gamma +1\right)}{\gamma (\gamma +2)}\right]\\ < & -\sqrt{n}{\left[\gamma (\gamma +2)\right]}^{-\frac{3}{2}}\left[\gamma +1-\frac{3\gamma (\gamma +1)}{\gamma \left(\gamma +2\right)}\right]\\ < & 0. \end{array}$$

The first inequation holds because:

(A5) $$\begin{array}{cc} \gamma +1+\frac{1}{\gamma +1} & -\frac{3\left(\ln\left(1+\gamma \right)-\frac{k\ln2}{n}\right)\left(\gamma +1\right)}{\gamma (\gamma +2)}\\ \; & >\gamma +1+\frac{1}{\gamma +1}-\frac{3\ln(1+\gamma )(\gamma +1)}{\gamma (\gamma +2)}\\ & >\gamma +1+\frac{1}{\gamma +1}-\frac{3\gamma +1}{\gamma +2}\\ & >\gamma +2+\frac{4}{\gamma +2}-4\\ & >0, \end{array}$$

which applies the inequality of the arithmetic and geometric means (AM-GM inequality) in the last step.

Last but not least, we have:

(A6) $$\begin{array}{cc} G_{n\gamma }^{{}^{\prime \prime }} & ={\left[n\gamma (\gamma +2)\right]}^{-\frac{3}{2}}\left[\frac{n\gamma (\gamma +2)}{2}+k-\frac{n\ln(1+\gamma )-k\ln2}{2}\right]\\ \; & >{\left[n\gamma (\gamma +2)\right]}^{-\frac{3}{2}}\left[\frac{n\gamma (\gamma +2)}{2}-\frac{n\ln(1+\gamma )}{2}\right]\\ \; & >{\left[n\gamma (\gamma +2)\right]}^{-\frac{3}{2}}\left[\frac{n\gamma (\gamma +2)}{2}-\frac{n\gamma }{2}\right]\\ \; & >0. \end{array}$$

In conclusion, we have the expressions of the derivatives of G and proved whether each of the derivatives is positive or negative.
